# I-Corps@NCATS trains clinical and translational science teams to accelerate translation of research innovations into practice

**DOI:** 10.1017/cts.2020.561

**Published:** 2020-12-07

**Authors:** Kathryn Nearing, Julie Rainwater, Stacey Neves, Pamela Bhatti, Bruce Conway, Nathaniel Hafer, Kevin Harter, Nicholas Kenyon, Margaret M. McManus, Demetria M. McNeal, Elaine H. Morrato, Suhrud M. Rajguru, Molly Wasko

**Affiliations:** 1 Division of Geriatric Medicine, School of Medicine, University of Colorado Anschutz Medical Campus, Aurora, CO, USA; 2 University of California, Davis, CA, USA; 3 School of Electrical and Computer Engineering, Georgia Institute of Technology, Atlanta, GA, USA; 4 Robertson Therapeutic Development Fund, The Rockefeller University, New York, NY, USA; 5 Center for Clinical and Translational Science, Program in Molecular Medicine, University of Massachusetts Medical School, Worcester, MA, USA; 6 Medical Innovation, Penn State University, State College, PA, USA; 7 Pulmonary, Critical Care, and Sleep Medicine, University of California, Davis, CA, USA; 8 Division of General Internal Medicine, School of Medicine, University of Colorado Anschutz Medical Campus Aurora, CO, USA; 9 Health Systems Management and Policy, Colorado School of Public Health, Innovation Ecosystem Program, Colorado Clinical and Translational Sciences Institute, University of Colorado Anschutz Medical Campus, Aurora, CO, USA; 10 Parkinson School of Health Sciences and Public Health, Loyola University, Chicago, IL, USA; 11 Departments of Biomedical Engineering and Otolaryngology, University of Miami, Miami, FL, USA; 12 Research, Innovation & Entrepreneurship and Faculty Success, Collat School of Business, University of Alabama at Birmingham, Birmingham, AL, USA

**Keywords:** Innovation Corps (I-Corps), training, innovation, evaluation, research commercialization

## Abstract

**Introduction:**

A key barrier to translation of biomedical research discoveries is a lack of understanding among scientists regarding the complexity and process of implementation. To address this challenge, the National Science Foundation’s Innovation Corps™ (I-Corps™) program trains researchers in entrepreneurship. We report results from the implementation of an I-Corps™ training program aimed at biomedical scientists from institutions funded by the National Center for Advancing Translational Sciences (NCATS).

**Methods:**

National/regional instructors delivered 5-week I-Corps@NCATS short courses to 62 teams (150 individuals) across six institutions. Content included customer discovery, value proposition, and validating needs. Teams interviewed real-life customers and presented the value of innovations for specific end-users weekly, culminating in a “Finale” featuring their refined business thesis and business model canvas. Methodology was developed to evaluate the newly adapted program. National mixed-methods evaluation assessed program implementation, reach, effectiveness using observations of training delivery and surveys at Finale (*n* = 55 teams), and 3–12 months post-training (*n* = 34 teams).

**Results:**

Innovations related to medical devices (33%), drugs/biologics (20%), software applications (16%), and diagnostics (8%). An average of 24 interviews was conducted. Teams reported increased readiness for commercialization over time (83%, 9 months; 14%, 3 months). Thirty-nine percent met with institutional technology transfer to pursue licensing/patents and 24% pursued venture capital/investor funding following the short courses.

**Conclusions:**

I-Corps@NCATS training provided the NCATS teams a rigorous and repeatable process to aid development of a business model based on customer needs. Outcomes of this pilot program support the expansion of I-Corps™ training to biomedical scientists for accelerating research translation.

## Introduction: The development of I-Corps@NCATS program

In April 2017, the National Center for Advancing Translational Sciences (NCATS) funded a 2-year supplement to develop the I-Corps@NCATS program (NIH GRANT UL1TR001417). The goal of the supplement was to develop and disseminate a training modeled on the National Science Foundation’s (NSF’s) Innovation Corps (I-Corps™) program [1,2]. The I-Corps@NCATS program aimed to engage clinical and translational researchers in the designing-for-dissemination and commercialization process from idea generation to practical (market) application. Specifically, I-Corps@NCATS provided teams of biomedical researchers, clinicians, and engineers across the career arc from undergraduate STEM (science, technology, engineering, and mathematics) students to senior scientists with entrepreneurial training to accelerate the translation of research discoveries into clinical and community-based practice. The specific aims of the I-Corps@NCATS supplement were to: 1) develop a uniform curriculum to be considered part of the official I-Corps™ body of knowledge and tailored to the commercialization of clinical and translational research discoveries in life sciences; 2) build capacity across Clinical and Translational Science Award (CTSA) hubs to deliver the standardized I-Corps@NCATS curriculum with fidelity through a regional train-the-trainer model; and, 3) establish a common evaluation framework, including program monitoring metrics; short-, intermediate, and long-term outcomes; and, field-tested instruments to assess the effectiveness and impact of the I-Corps@NCATS program across CTSA sites. The following CTSAs served as regional training hubs: University of California Davis (UC Davis), University of Colorado Anschutz Medical Campus, University of Alabama at Birmingham (UAB)/Georgia CTSA, University of Miami, Penn State University, and University of Massachusetts Medical School. Michigan and Rockefeller were also members of the I-Corps@NCATS development team. These partner institutions relied on local relationships for regional programs but helped develop the curriculum and shared lessons learned from teaching I-Corps™ in their life-sciences contexts.

To create the original I-Corps™ program in 2011, NSF brought together innovative ideas from business, including methods of customer discovery [3] and “lean” ideas of agile business development [4]. Customer discovery occurs as a rapid, immersive process that involves directly interacting with potential customers through interviews and site visits to observe and operationalize how a given activity or function is currently performed. Teams document relevant workflows, which help research teams think about who the customers are. This rapid ethnography of immersing oneself in translational contexts, and directly interacting with those one hopes will ultimately benefit from an intervention or innovation, is done early and often. Brief (usually 20 minute) interviews explore customer “jobs to be done;” how these responsibilities/needs are currently achieved or fulfilled; pains (challenges, gaps, tensions); and, gains (possible benefits, rewards, motivations, and incentives, for example, to do things differently) [4,5]. Importantly, teams do not use interviews as a time to pitch their ideas but rather to invest in learning about relevant contexts in order to optimize problem-solution-fit. Resulting insights from interviews are organized into a value proposition canvas [4] – a subset of the business model canvas [6] – that helps investigators articulate a value proposition that is aligned with their customer segment and stakeholders. The value proposition and business model canvas evolve over the course of successive waves of interviews and iteratively inform priorities for product features and/or “pivots” toward a strategic, nuanced market niche [5]. I-Corps™ teams receive coaching support throughout this process from the teaching team who pose questions to generate reflective appraisal of the information surfacing from interviews and to challenge teams to avoid confirmation bias. Thus, design decisions are no longer made in a vacuum or isolated from the clinical/community-based settings and end-users of translation, but rather are data-informed through a rigorous, repeatable methodological approach. Furthermore, the investment in learning from and about end-users, and the ecosystem that will influence decisions to adopt a given innovation, is done early – in many cases, during the design/conceptualization phase. The I-Corps™ methodology resonates with clinical and translational researchers because the underlying process helps generate and test emerging hypotheses regarding the value of their innovation. Teams explore the validity of their value proposition with key decision makers who, based on their role/position in an organization or within the workflow, may influence the eventual adoption of an innovation. In this way, clinical and translational scientists are encouraged to get out of the lab and off campus, to learn about relevant aspects of the contexts in which their innovation may be introduced. “Go/no go” decisions regarding whether further investment in an innovation is warranted also occurs relatively early in the research and development process, allowing clinical and translational research teams to redirect effort toward other endeavors.

**Fig. 1.**
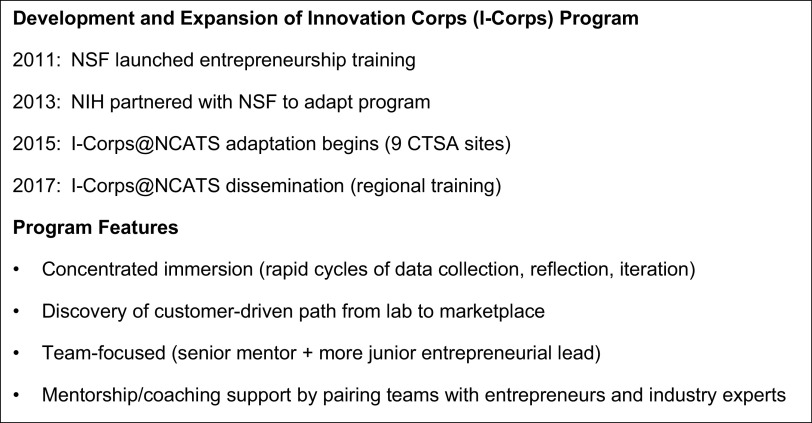
Innovation Corps (I-Corps) program development and key features.

NIH adapted the NSF I-Corps™ entrepreneurial training program for life-science researchers to help bridge the so called “valley of death” – the schism between research development and market application. The NIH SBIR (Small Business Innovation Research) and STTR (Small Business Technology Transfer) grant programs serve as pipelines for clinical and translational researchers to access I-Corps™ training through the I-Corps™ at NIH program [1]. This created a gap for researchers in life sciences interested in exploring business potential before creating an SBIR-/STTR-funded business. To address this gap, the national network of nearly 60 CTSA programs, funded by NCATS, offered another dissemination network to link a broader spectrum of clinical and translational research teams to entrepreneurial training. For NCATS, I-Corps™ represented another tool to promulgate the acceleration of research translation from the lab to clinical practice. While biomedical research institutions and teams were seeding and creating tremendous innovation, more often than not, they lacked bi-directional connectedness to industry [7]. I-Corps™ infuses entrepreneurial thinking into the clinical and translational sciences, creates a structure or mechanism for catalyzing connections between industry and the innovations emerging from academia, and challenges researchers to get out of the building and off campus to network and build connections of their own. Fig. 1 provides a summary of the program dissemination timeline and program features.

## Materials and Methods

### I-Corps@NCATS Teams

**Fig. 2.**
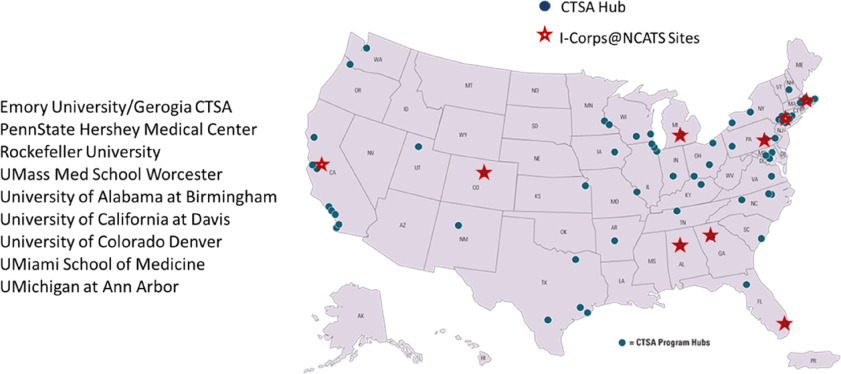
I-Corps@NCATS supplement sites.

A total of eight cohorts, comprised of 62 teams and 150 individuals, completed the regional I-Corps@NCATS short course during the 2-year supplement (Fig. 2). Typically, participating teams consisted of a senior investigator or clinician and a graduate student, a post-doctoral trainee/fellow, a resident, or early career investigator. The relatively more junior member of the team served as the “entrepreneurial lead” who assumed the primary responsibility for coordinating and conducting customer discovery interviews, presenting key insights and implications for the team’s value proposition, and investing the significant time necessary to capitalize on the momentum created during the I-Corps@NCATS training. We categorized teams based on the type of innovation they were pursuing, such as the development of a drug or biologic, medical device, software application, or diagnostic tool. Table 1 shows the number and types of teams participating in the I-Corps@NCATS regional short courses during the supplement. Importantly, participating teams represented the full spectrum of clinical and translational research. During the supplement, the expertise and innovations of I-Corps@NCATS teams extended beyond life sciences to include those seeking to promote a research service or educational product to enhance clinical and translational research capacity. Key learnings and team successes, as well as the presence of successive cohorts on campus, fostered peer mentorship. Former participants volunteered to serve as guest panelists to share experiences and answer questions, or as industry mentors for current teams. Teams participating as part of the same cohort benefited from hearing peers present the results of their customer discovery interviews.

**Table 1.** Number and types of teams that participated in I-Corps@NCATS regional short courses

	UAB/Georgia Tech	University of Miami	University of California Davis	University of Colorado Anschutz Medical Campus (Spring)	University of Colorado Denver (Fall)	U-Mass	Penn State University
**Short Course Kickoff to Finale**	Jul 2018 to Aug 2018	Aug 2018 to Aug 2018	Sep 2018 to Oct 2018	April 2018 to May 2018	Oct 2018 to Nov 2018	Oct 2018 to Nov 2018	Jan 2019 to Mar 2019
**Teams registered (# completing)**	16 (9)	14 (7)	13 (10)	9 (8)	10 (10)	12 (7)	11 (11)
**Type of team completing short course**							
**Medical Device** (*n* = 18)	0	1	3	1	4	3	6
**Drug/Biologic** (*n* = 11)	1	1	4	0	0	2	3
**Software Application** (*n* = 9)	3	1	2	2	1	0	0
**Research Services** (*n* = 6)	2	0	1	1	1	0	1
**Diagnostic** (*n* = 5)	1	1	0	0	3	0	0
**Other (e.g., education;** *n* = 6**)**	0	0	0	4	1	1	0
**Not Available** (*n* = 7)	2	3	0	0	0	1	1
**Total Individuals Completing I-Corps@NCATS**	**21**	**14**	**27**	**34**	**13**	**20**	**21**

### I-Corps@NCATS Training Program

The 5-week I-Corps@NCATS short course involved at least two full-day in-person sessions (a “Kick-off” and “Finale”), during which participants received didactic presentations on core concepts delivered by national and regional instructors. Examples of core concepts include “pains,” “gains,” and “jobs to be done” – concepts that emphasized the importance of understanding customers’ day-to-day roles, as well facilitators and barriers to fulfilling those roles or expectations. Teams received books [4–6,8], links to videos, and other resources to build their background knowledge and enhance access to tools/templates, such as the business model canvas. Teams also engaged in hands-on activities to practice the skills needed to identify and prioritize key customer segments and conduct 20–30 customer discovery interviews – an explicit goal for teams between the Kick-off and Finale.

Given the complexity of clinical and translational research (specifically, the myriad of stakeholders at different levels, the complex regulatory environment, and often competing incentives), instructors dedicated significant time during the Kick-off to the topic of customer segmentation. For example, participants practiced operationalizing each step in a clinical workflow and identified who might be impacted by the implementation of a specific innovation. This exercise underscored for participants the contingencies and ripple-effects created by introducing a new approach or technique within a system, as well as vested interests in maintaining the status quo. Instructors emphasized the importance of operationalizing the “value chain” and the various stakeholders represented, including patients, providers, healthcare system administrators, regulatory specialists, and policy makers. During the short course, instructors coached teams to focus on two primary customer segments: end-users and buyers (i.e., those in an organization or group with the authority make purchasing decisions). Instructors provided strategies regarding ways to network and reach representatives of these customer segments and actively leveraged their own networks, including the CTSA network, to connect teams with individuals to interview.

In between the Kick-Off and Finale, teams conducted customer discovery interviews and participated in “Office Hours” (i.e., coaching sessions) to process what they were learning and the implications for their value proposition. Teams also participated in a videoconference midpoint meeting during which each team presented their progress and emerging insights. Teams received a templated slide deck to guide the development of presentations given during the virtual midpoint meeting, as well as during the Kick-Off and Finale. Formal presentations described the team, proposed and actual customer discovery process, hypothesized value propositions and emerging business model. Serial entrepreneurs, representatives of technology transfer offices, and members of instructional teams provided coaching support as teams presented their work.

### Train-the-Trainer Model to Establish Instructional Teams

Instructional teams delivered the I-Corps@NCATS curriculum at each regional program. Instructional teams included a national trainer, the site principal investigator (PI), and at least one PI from another regional training hub (e.g., the site PI from UC Davis participated as a member of the instructional team at UAB). The four national instructors were serial entrepreneurs with extensive industry experience and networks, had served as national I-Corps™ trainers for NSF and I-Corps™ at NIH, and brought relevant experience in the clinical and translational sciences, including development and commercialization of medical devices and regulatory consultation in the life sciences.

Site PIs who served as regional trainers during the supplement held multiple leaderships roles at major biomedical research institutions and in local entrepreneurial ecosystems; they, thus, served to bridge academia and industry. Site PIs advertised the program, recruited cohorts of participants, and worked with instructional teams to deliver the curriculum and coaching support throughout the short course. Instructors (both national and regional) participated in regular conference calls throughout the supplement to coordinate implementation efforts, share best practices, and problem-solve issues, as needed.

### Program Evaluation

A core objective of the I-Corps@NCATS supplement was to develop the methodology to evaluate the newly adapted program and its implementation in a CTSA context. The I-Corps@NCATS program was formally evaluated by a national team representing three evaluation professionals with extensive CTSA evaluation experience affiliated with the UC Davis Health Clinical and Translational Science Center and the Colorado Clinical and Translational Sciences Institute (CCTSI). To determine the degree to which specific aims of the supplement were achieved and to inform program expansion to additional CTSA sites, the evaluation plan focused on assessing:

1. Fidelity of Implementation and Sustainability: The degree to which core components of the newly developed I-Corps@NCATS curriculum were implemented uniformly across participating sites and the potential to sustain and disseminate the I-Corps™ program across the CTSA consortium;
2. Participant Experience: Satisfaction with I-Corps@NCATS program content and delivery, as well as anticipated facilitators and barriers to commercialization; and,
3. Pathways to Success: Immediate post-training and 3–6 months intermediate outcomes relevant to I-Corps™ goals.

Specific methods used in relation to each of these evaluation domains are described in detail below and are summarized in Table 2.

**Table 2.** Summary of I-Corps@NCATS Evaluation Instruments

Evaluation Focus	I-Corps@NCATS Evaluation Tools	Description
**Participant Experience and Pathways to Success**	**Post Survey**: Survey of all participants to assess satisfaction, stage of readiness for commercialization, collect Net-Promoter score, potential next steps	Participants completed the survey immediately following the training Finale
		Includes 45 teams from all sites
		Response rate: 78%–100%
	**Non-Completers’ Survey:** Survey of those teams lost to attrition between Kick-off and Finale	Explored reasons for not completing the short course, with specific focus on fit and feasibility
		Includes 8 teams from UAB, Miami, UC Davis
		Response rate: 43%–66%
	**Longitudinal Survey:** Survey of team leaders to explore intermediate outcomes	Team leads completed the survey 3–6 months following the training Finale
		Includes 35 teams from UAB, Miami, UC Davis, Colorado
		Response rate: 57%–100%
**Fidelity of Implementation**	**Fidelity Checklist and Observational Protocol:** Standardized template for documenting observations and assessing fidelity (i.e., delivery of core training components)	Used by evaluators to collect observations at Kick-off and Finale; direct observations were supplemented by collecting, reviewing, and archiving program artifacts
**Sustainability**	**Facilitated Discussion Guide:** Virtual focus groups with national and regional instructors	Facilitated discussions were conducted 1–2 months following trainings to give key informants a time to reflect on their experience with program implementation

#### Fidelity of implementation and sustainability

To assess fidelity of implementation of core training components, evaluators observed each Kick-off, completing a detailed observation form and fidelity checklist (Supplemental Material). With one exception (UMass), at least one I-Corps@NCATS evaluator attended each of the regional trainings to conduct direct observations of Kick-Off days and complete detailed field notes. Observations were completed by the local evaluator at UMass (McManus) after being oriented to the observational protocol and fidelity checklist by a national evaluator (Nearing). Evaluators supplemented direct observations with reviews of program artifacts (e.g., national instructor slide decks, program agendas, team applications, and program rosters). All national and regional instructors were engaged in virtual focus groups during which they reflected on program implementation and identified issues relevant to feasibility and sustainability, including implications for scaling the program for national dissemination. Evaluators used these facilitated discussions as another opportunity to explore similarities and differences across sites in terms of training content and structure/organization, as well as in the teams who participated. (Facilitation guides are included as Supplemental Material.)

#### Participant experience and pathways to success

Teams completed surveys designed to collect information immediately following the training (at the conclusion of each site’s Finale) and 3–6 months following program completion. The **Post-Training Survey** (Supplemental Material) was administered to all participants of teams who completed the training and asked about the team’s experience (i.e., program satisfaction), customer discovery interviews (number completed and impact on overall experience), challenges, perceived commercialization readiness, and planned next steps. Evaluators also administered a brief **Non-Completers’ Survey** (Supplemental Material) to those teams that registered but did not complete the short course. This survey explored reasons for attrition and solicited feedback about aspects that might have made program completion more feasible. Finally, evaluators developed a **Longitudinal Follow-up Survey** (Supplemental Material), which was administered in February 2019 to team leads from all sites but UMass and Penn State, as fewer than 3 months had passed since the Finale at these sites (November 20, 2018 and February 15, 2019, respectively). The development of the Longitudinal Follow-up Survey was informed by (1) longitudinal follow-up interviews conducted with two cohorts of Colorado-based teams in the first year of the supplement, (2) NSF’s *I-Corps™ Longitudinal Outcomes Survey* [9], and (3) CTSA common evaluation metrics. The survey explored intermediate and long-term outcomes such as reconstituting teams to fill identified gaps in knowledge and skills; new customers identified; expanded networks; prototype development; new marketing approaches; changes in career/academic trajectories; intentions to participate in the national I-Corps™ program; SBIRs/STTRs submitted and awarded, invention disclosures, patents filed and approved (national and international); publications; and, private and public funding investment. Skip logic was used to explore a variety of outcomes and commercialization pathways. The longitudinal survey also featured open-ended items that investigated progress in the team leaders’ own words to help identify the diverse array of possible outcomes. Both the post-training and longitudinal survey featured a *Net Promoter Score* question, which is a widely used indicator of potential demand for a program. All surveys were administered electronically using Qualtrics version XM (Qualtrics, Provo, Utah). Team survey response rates ranged from 78% to 100% for the post-training survey. Response rates for the three sites included in the longitudinal survey ranged from 57% to 100%. Response rates to the non-completer survey ranged from 43% to 66%. Table 3 details this variation by site and survey. The Non-Completers’ Survey was not administered in Colorado, UMass, or Penn State as these sites experienced no attrition between the Kick-Off and Finale at their respective sites.

**Table 3.** Survey Response Rates by I-Corps@NCATS Site

Site	Teams registered	Teams completing short course	Post-surveys completed (%)	Longitudinal surveys completed (%)	Non-completer teams surveyed (%)
UAB/Emory/Georgia Tech	16	9	7 (78%)	7 (78%)	3 (43%)
University of Miami	14	7	6 (86%)	4 (57%)	3 (43%)
University of California Davis	13	10	9 (90%)	10 (100%)	2 (66%)
University of Colorado (Spring)	9	8	6 (75%)	6 (75%)	N/A
University of Colorado (Fall)	10	10	10 (100%)	7 (70%)	N/A
U-Mass	12	7	7* (100%)	N/A	N/A
Penn State University	11	11	10 (91%)	N/A	N/A

* Eight responses were submitted to the survey, one team had two responses.

## Results

### Number and Types of Teams

Of 85 teams registering, 62 teams (73%), comprised of 150 individuals, completed the I-Corps@NCATS regional short courses. Teams were pursuing diverse innovations, including the development of medical devices (33%), drugs/biologics (20%), software applications (16%), research service innovations (9%), diagnostics (8%), or other products such as educational innovations/services (11%). (Supplemental material features examples of innovations represented by I-Corps@NCATS teams, including details regarding stage of development and phase of the clinical and translational research spectrum, as well as milestones achieved in their respective commercialization pathways. We also provide a narrative case example of the impact of I-Corps training for one Colorado-based team and their journey to bring a medical device to the marketplace.) Most teams that did not complete the short course reported lack of adequate time for the intensive 5-week training as the reason, rather than lack of perceived value in the course or process.

### Customer Discovery Interviews

Teams completed an average of 24 customer discovery interviews during the 5-week short courses. When asked to rate the importance of the various components of the short course, the customer discovery process was rated by all teams as very important – an average of 4.7 on a 5-point scale (Table 4).

**Table 4.** Average number of interviews and importance of the customer discovery process* (scale: 1 = not at all important; 5 = extremely important)

	UAB	UC Davis	Colorado (Spring ’18)	Colorado (Fall ’18)	U-Mass	Penn State	AVE
Average # of interviews per team	18	41***	26	28	14	19	**24**
Importance of customer discovery interviews**	4.8	4.3	4.8	4.8	4.7	5.0	**4.7**

* Data Source: Post-Survey, validated by information reported by teams during Finale (documented in PowerPoint slides).

** Item not included in Miami survey.

*** One team at UC Davis conducted 150 interviews; Mean = 22 without this outlier.

### Longitudinal Outcomes

**Fig. 3.**
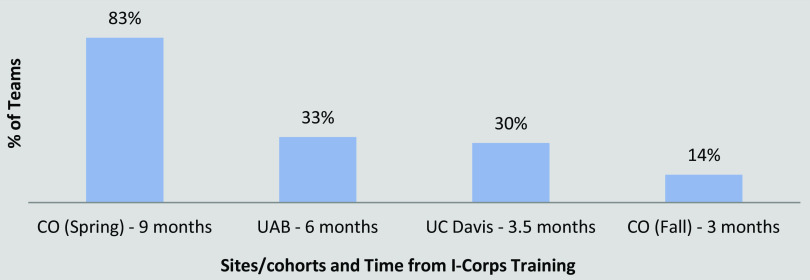
Percentage of teams that reported being ready for commercialization at follow-up.

The longitudinal follow-up survey asked teams to describe the activities they had completed since the Finale. Most teams (over 80% on average) reported that they continued to meet regularly as a team and continued the customer discovery process. Over a third (39%) of respondents reported meeting with tech transfer at their local institution to pursue licensing and patents. Finally, 24% of respondents indicated that their team pursued venture capital/an investor. An increasing percentage of teams reported readiness for commercialization over time (83%, 9 months after course completion; 14%, 3 months after course completion; Fig. 3).

### Challenges to Commercialization

At follow-up, teams reported the barriers and challenges they experienced in implementing a successful business model. Over half reported needing both industry-based and academic or university-based mentorship to advance their efforts (55%). Almost half of teams said they had challenges identifying commercial mentors and partners (45%). Over a third of teams also reported challenges with securing funding for technical validation and proof of concept (39%) and in putting together a financial business model or plan (35%).

### Participant Experience

The majority of participants were enthusiastic about the I-Corps@NCATS short course. They indicated that the training had given them a better understanding of their value proposition and how to position their product strategically within a given market segment and among competitors. The Net Promoter Score question on post-training and longitudinal follow-up surveys asked respondents, on a scale of 1–10, how likely they were to recommend the I-Corps@NCATS program to a friend or colleague who is interested in commercializing or sustaining the impact of their innovation. At both the conclusion of the training and follow-up, I-Corps@NCATS Program “promoters” (those responding 9 or 10) outnumbered the “passives” (those selecting a 7 or 8) and “detractors” (0–6). Further, average Net Promoter Scores increased over time from 60 to 69 – a finding that underscores the durability of the learning and the applicability to other projects.

### Fidelity of Implementation

Overall, the I-Corps@NCATS program was delivered in a uniform manner. A small cadre of experienced national instructors delivered most of the core curriculum and coaching at newly adopting sites; Denver – a regional hub with an established record of providing I-Corps™ trainings twice annually – utilized a regional instructional team representing multiple universities and campuses in Colorado. Evaluation documented that each training covered the key core concepts outlined in the standardized Observational Protocol and Fidelity Checklist. Some variation was noted across participating hubs in training structure (e.g., not every site offered an introductory webinar or incorporated Office Hours at the conclusion of the Kick-Off), the degree to which trainings incorporated experiential components, and some of the resources used.

### I-Corps@NCATS National and Regional Instructor Feedback

Discussions during two virtual focus groups with national and regional instructors highlighted important considerations for sustaining the program at regional sites and expanding the program through the CTSA network. Both depend on providing personnel and resources at this stage. Local instructors emphasized that the process of recruiting appropriate teams for successive cohorts was the most resource-intensive aspect of program implementation. They also noted that the network created among the instructors was an essential structure to support fidelity of implementation, as well as sharing strategies and solutions to enhance feasibility. Instructors suggested that asynchronous learning modules may be a solution to consider in order to make didactic components more widely available to CTSA investigators, support recruitment and provide an initial orientation to the course, leaving relatively more time during in-person workshops to practice applying new knowledge/skills and receive coaching support. Site PIs noted the need for funding for teams who wish to continue customer discovery interviews and pursue additional training opportunities, such as the more extensive national I-Corps™ program.

### Conclusions

Evaluation findings indicate that sites collectively accomplished the aims of the supplement: to develop a uniform I-Corps™ curriculum, appropriately tailored for clinical and translational investigators, and delivered with fidelity across participating sites that spanned the nation and unique entrepreneurial ecosystems. The train-the-trainer model supported capacity building and fidelity of implementation. Trainings attracted diverse teams, with products representing all segments of the translational spectrum, who consistently expressed high satisfaction with the quality and value of the course. Most teams continued to meet, conduct customer discovery interviews, and engage with coaches following the training.

In addition to the instructional and entrepreneurial capacity established during the supplement, evaluation capacity was also achieved. Specifically, national evaluators, in consultation with national and regional instructors, delineated appropriate outcomes; developed, piloted, and refined instruments for tracking teams’ highly contingent commercialization pathways; and, provided preliminary evidence regarding program impact. We learned that program participation can fundamentally change the way clinical and translational investigators think about their research and how they might optimize translational impact. As one participant stated:

[Academics are] really comfortable on the front end. I can set up a nice test – RCT [to] test efficacy. I feel really comfortable with that. Often, we stop there in academia, and so [I-Corps™ has] really opened up the back end – the dissemination piece – and how we can actually make this more scalable and be thoughtful about [that] even before the train leaves the station, if you will. That is something that I-Corps™, I think, has really helped.

This finding – that I-Corps@NCATS can enhance translational and entrepreneurial thinking – has profound implications for longer-term outcomes related to grant writing, research productivity and impact, academic and career trajectories, teaching and mentorship. The evaluation also highlighted that commercialization, not unlike other types of translation, takes time. Specifically, the proportion of teams who reported that they were ready for commercialization increased incrementally over the first few months following program participation. Still, other teams realized relatively quickly that they needed to make a “no go” decision – another positive outcome of I-Corps@NCATS, which gives teams the tools and techniques to “fail fast” before investing time and monetary resources in either a) developing a research solution for a problem that does not exist (from the perspective of the user) or, b) if the problem does exist, investing in the development of a solution that is not a good fit.

For teams moving forward with the product that was the focus of the I-Corps@NCATS short course (and perhaps even for teams that made a “no-go” decision but now have gained an entrepreneurial mindset and tools they can apply to other endeavors), mentorship support, particularly from industry, was critically important and a major need. Longitudinal survey data suggest that teams lack industry mentorship and commercial connections even though many remain connected to I-Corps@NCATS instructors. Because they are based in academic biomedical research institutions, CTSAs may struggle to establish relationships with industry. Yet, not having connections to industry can negatively impact translation. While very few teams were ready to pitch their ideas to industry, such connections might facilitate networking to continue the customer discovery process and provide access to other types of resources, including locations for field testing innovations and connections to future investors [8]. The finding regarding the primacy placed on industry-based mentorship by participants underscores the need to build such connections with industry as part of a robust translational and entrepreneurial ecosystem in which these teams and the I-Corps@NCATS program can thrive. Understanding the value of industry connections for I-Corps@NCATS teams – what they are seeking from these relationships, what resources these relationships provide that would not otherwise be available, and the effect on commercialization – is an important area on which to focus longitudinal evaluation in the future. Results can inform strategic investments in the purposeful establishment of industry-based mentorship for successive cohorts of I-Corps@NCATS teams. Importantly, such efforts would also help teams prepare to participate in the national I-Corps@NCATS program, which requires industry mentorship.

It is noteworthy that during the supplement no teams applied to the national NSF I-Corps™ program, even though teams that had completed I-Corps@NCATS were eligible. Site PIs noted, however, that teams from their sites, who completed the short course previously, had gone on to participate in the national NSF program. Taken together, these findings suggest that teams completing the course during the supplement may not have matured to the point that they were ready for the national NSF program. It may also be that, while the short course has been successfully adapted for clinical and translational scientists and the context of CTSAs, a similar effort to tailor the national program for this audience is needed to enhance acceptability, accessibility, and participation. In processing the finding regarding lack of participation in the national program, site PIs and instructors noted the following aspects of the national I-Corps™ at NIH program that make it difficult for researchers, clinicians, and trainees in life sciences to participate:

1. The I-Corps™ at NIH program specifically supports SBIR-STTR awardees, which must have established a company structure. Establishing a company has not been a benchmark of success for biomedical researchers who are challenged, instead, to maintain continuous grant funding and produce peer-reviewed publications in high-impact journals. Furthermore, starting a company may have even less salience for teams working at different phases of the translational research spectrum; for example, “translation to population” teams may be looking for non-profit sustainability models for their health interventions. Also, teams working on drugs, devices, and diagnostics may define success as out-licensing, rather than creating a company, given that out-licensing has been a model more commonly pursued by university-based technology transfer offices [10]. This misalignment of incentives may mean that the I-Corps™ at NIH national program has less perceived value (problem-solution-fit) for many scientists.
2. While I-Corps@NCATS teams were eligible to participate in the NSF I-Corps™ National program, we found that the 7-week duration of the NSF National I-Corps™ program, and the 100+ customer interview requirement, may not be feasible for biomedical researchers. Medical campuses are more likely to have a “soft money” academic funding model and 12-month academic appointments. Consequently, faculty compensation and promotion are dependent upon generating clinical revenue and/or funding from federal grants. Teams may have to plan a semester or more in advance as they juggle clinical, research, and administrative responsibilities with other teams and administrative units.

Exploring ways to tailor the I-Corps™ at NIH program for clinical and translational scientists and teams may be an important future direction to consider. Site PIs and instructors noted strategies to help bridge the short course and the national NIH program. For example, some sites incorporated seed grants into their CTSA programs to provide teams that had successfully completed the short course with additional funding to continue the customer discovery process and to travel to professional meetings and trade shows to network and initiate industry-based mentorship relationships. Site PIs and instructors also noted the importance of having team members at an earlier stage of their careers. Graduate students, trainees and early-career investigators may be more agile in terms of their ability to carve out time and may be more willing to consider alternative career paths, with the latter serving as a major source of motivation to invest in attending the national training program. One site PI hypothesized that “the more we can engage trainees, the more likely we are to see teams go to the national program.” The site PI called this “flow through talent,” referring to the person on the team who may be less invested in pursuing a more traditional academic research trajectory and, therefore, willing to assume a leadership role (and the associated risk inherent) in starting a company.

## Building on Lessons Learned to Grow the I-Corps@NCATS network through CTSA Hubs

Building upon the successful development and dissemination of the I-Corps@NCATS pilot program, in 2020 the University of Alabama at Birmingham CTSA hub received supplemental funding to expand the I-Corps@NCATS program through a CTSA Competitive Revision award, grant number 3UL1TR003096-02S1. Leveraging the nine CTSA hubs participating in the pilot as mentor sites, we will train an additional 13 CTSA Hubs over the next 3 years to bring the total number of I-Corps@NCATS-trained CTSA hubs to 22 – over a third of the CTSA hub network. The following CTSA hubs are serving as mentee sites: Case Western Reserve, Columbia University, the Medical College of Wisconsin, the Medical University of South Carolina, Northwestern University, Oregon Health & Science University, Rutgers University, University of Buffalo, University of Chicago, University of Rochester, University of Texas – Medical Branch, and the University of Virginia. Building upon the lessons learned featured in this paper, we will integrate the following program enhancements into future I-Corps@NCATS program offerings as we seek to further expand I-Corps@NCATS within the CTSA context:

- We will increase the reach of I-Corps@NCATS across CTSA institutional boundaries by offering online programs. This past year, all I-Corps™ national programs have had to quickly pivot to a completely online format. For at least the first year of implementation, I-Corps@NCATS will also be offered online. This has the potential to significantly expand access to the program for potential teams, while reducing costs associated with travel. The online format enables us to accept teams from any CTSA hub, as well as include additional CTSA hubs as observer/mentee sites.
- We will expand access to the resources needed for staffing the I-Corps@NCATS training program by connecting with each CTSA hub’s unique translational and entrepreneurial ecosystems. Personnel are needed to enhance site PI capacity to recruit teams; disseminate information regarding the program (e.g., benefits/opportunities of participation, as well as the time commitment); and, to connect teams to industry mentors. Many of our participating CTSA hubs are linked to existing I-Corps™ resources, including NSF I-Corps™ sites, regional nodes, and state-run programs, such as I-Corps@Ohio, although in many instances these programs are at best only loosely connected. By working across institutional internal and external boundaries, I-Corps@NCATS participation will help build local entrepreneurial ecosystems by connecting I-Corps™ activities among NIH funded life sciences, NSF funded STEM innovations, local innovation centers, and industry partners.
- To sustain high-level fidelity, we will maintain a structure for connection and collaboration across I-Corps@NCATS sites to build a national community of practice to share insights and lessons learned. This will also allow us to better leverage the CTSA network to facilitate I-Corps@NCATS teams’ engagement in customer discovery. Clinical and translational scientists need to better understand how things work in hospital settings, for patients, nurses, and surgeons, and the CTSA hubs are the place to find people to interview. This is an essential value-add of creating synergies between the CTSA hub network and I-Corps™ programs to accelerate the translation of biomedical discoveries into improved patient care.
- Finally, we will disseminate evaluation products (instruments, report templates, logic model) with the program model to standardize data collection and reporting. This will support the ability to aggregate process and outcome measures across teams and CTSA sites to assess collective capacity for consistent, effective program delivery and impact.

Over the next 3 years, we aim to offer 21 I-Corps@NCATS short courses across 22 CTSAs, reaching over 3000 translational scientists throughout the career arc and accelerating the translation of over 600 biomedical innovations for improved patient care. With the addition of online programs, we are able to expand our reach to translational scientists across the CTSA hub network, including partner institutions that may not have access to local alternatives. Through I-Corps@NCATS, we aim to significantly accelerate the path from lab discovery to improved human health by ensuring our research teams not only conduct scientifically rigorous research, but also research that has demonstrated relevance to our healthcare communities and the potential for impact through the pathway of commercialization.

## Supplementary material

To view supplementary material for this article, please visit https://doi.org/10.1017/cts.2020.561.
